# Processing pro-drop features in heritage Turkish

**DOI:** 10.3389/fpsyg.2022.988550

**Published:** 2022-11-15

**Authors:** Serkan Uygun

**Affiliations:** Faculty of Educational Sciences, Department of English Language Teaching, Bahçeşehir University, Istanbul, Turkey

**Keywords:** heritage speakers, Turkish, pro-drop, subject-verb agreement, sentence processing, morphosyntactic knowledge

## Abstract

Previous studies have reported that null subject is not completely lost in heritage speakers, but there is an increase in the production and acceptance of overt subjects. Turkish is a pro-drop language and as a typical feature of pro-drop languages, it requires obligatory verb agreement marking for sentences with null subjects. However, Turkish subject-verb agreement marking is an example of optional agreement in which the 3rd person plural subject has optionality and can be used with singular verb forms under certain conditions. The current study investigates the reading times (RTs) of plural-marked and unmarked verbs in sentences with overt and null subjects during real time sentence processing in comparison to non-heritage speakers of Turkish via a self-paced reading experiment. Significant differences were observed between the heritage and non-heritage speakers of Turkish indicating both quantitative and qualitative real-time processing differences between the two groups. These differences suggest that Turkish heritage speakers need more time to integrate the information in real time processing.

## Introduction

The original version of the Interface Hypothesis (IH) predicts increased vulnerability for bilinguals in phenomena, where syntax interacts with other modules of language, known as the interfaces (Sorace and Filiaci, 2006). On the other hand, no problems/difficulties were expected for purely syntactic phenomena. The revised version of the IH (Sorace and Serratrice, 2009) made a linguistically principled distinction between internal and external interfaces. The internal interfaces mainly integrate modules that pertain to formal grammar, such as syntax, semantics, and morphology and their interactions (e.g., morphosyntax). On the other hand, external interfaces combine linguistic modules that are related to general cognition and/or world knowledge, like discourse and pragmatics. In other words, external interfaces involve interactions between linguistic and non-linguistic domains (e.g., syntax-pragmatics). According to this version, only external interfaces are expected to be particularly vulnerable/problematic because external interfaces integrate domains from different language levels, which leads to a higher processing load. Phenomena located at external interfaces are predicted to be vulnerable/problematic in bilingual populations either because of their less detailed knowledge or less automatic access to computational constraints within the language module, or because they have fewer cognitive resources available (Sorace, 2011). According to Tsimpli and Sorace (2006), the main difference between internal and external interfaces is that only the latter requires a higher level of language use because of integrating domains outside of the formal grammar. Sorace (2011) also claims that the IH makes explicit claims for the heritage speakers (HS) at the level of ultimate attainment. Therefore, the claims of IH can be applied to HS as they are an important testing ground for the IH (Montrul and Polinsky, 2011). HS are defined as individuals who were raised in homes where a language other than the dominant community language was spoken, resulting in some degree of bilingualism in both the heritage and the community language (Scontras et al., 2018). Recent studies with HS have suggested that they have control over the rules of particular modules (syntax, phonology), but they have difficulty when integrating grammatical and non-grammatical information (Benmamoun et al., 2013). While Sorace and Serratrice (2009) and Sorace (2011) advocate that structures that lie at the external interfaces are vulnerable/problematic for HS with their higher processing load, Benmamoun et al. (2013) questions whether other interfaces are also affected under heritage language conditions without making a distinction between internal and external interfaces. Their conclusions suggest that HS experience difficulties/problems when they have to compute interface properties. They observed that HS struggle with operations that involve computation across more than one grammatical component (e.g., syntax and morphology) because interface operations in general require knowledge of the principles and constraints operating on both components, together with the ways in which they map onto each other (Benmamoun et al., 2013, p. 165).

Psycholinguistic research on bilingualism has mainly focused on the representation and processing of structures that require the integration of knowledge from different linguistic domains for over 20 years. The appropriate use of some structures cannot be merely determined by syntactic rules, but requires the integration of knowledge from other domains. A typical example is the pro-drop phenomenon. Pro-drop is defined as the deletion of the overt subject in a sentence in cases when the subject may be recovered from the pragmatics and the context of the sentence or from the person information on the verb (Altan, 2013). This feature is usually seen in languages with a rich inflectional morphology. Pro-drop languages habitually use overt subjects mainly to mark pragmatic information such as contrast, emphasis or topic shift (e.g., Enç, 1986). In other words, the selection of the overt and null subjects requires both syntactic and discourse-pragmatic knowledge, which makes the pro-drop phenomenon relevant to the syntax-pragmatics interface, which is an external interface.

The majority of the previous studies with HS have examined the contact between the pro-drop and the non-pro-drop languages mainly focusing on the cross-linguistic influence. Studies on a variety of languages that allow both overt and null subjects (e.g., Italian, Greek, Spanish, and Russian) have reported that null subject expression is not completely lost in HS with the exception of severe cases of attrition, HS display an increase in the production and acceptance of overt subjects, they lose or display variability in the discourse-pragmatic constraints on overt subjects in the pro-drop language and they have a tendency to use overt subjects in pragmatically redundant contexts, for example, when a referent is not marked for contrast, emphasis or topic shift (Silva-Corvalán, 1994; Polinsky, 1995; Montrul, 2004; Tsimpli et al., 2004; Sorace, 2005; Albirini et al., 2011). According to Polinsky (2018), these results are not surprising, because overt subjects do not change much in the structure of a heritage language (HL). Overt subjects are not ungrammatical in the baseline and they allow HS to be clearer in production because HS exhibit a preference for one-to-one mapping between form and function. The overall use of overt subjects is consistent with the general tendency for overmarking observed in HL. In addition, several researchers have observed that the use of null subjects is already diminished in the speech of first-generation immigrants, whose language serves as input to HS (Otheguy et al., 2007; Montrul, 2016). The decreasing use of null subjects in the input also contributes to the erosion of null subjects in the HL. While most of the existing studies that have compared overt and null subjects have tested the use of overt pronouns, the present study focuses on the processing of sentences with overt subject noun phrases (NPs) and null subjects in Turkish HS.

### Pro-drop in Turkish

Turkish is a morphologically rich language in which verbs must agree with the subject in person and number, and the subject position of a sentence or a noun phrase does not need to be filled overtly with a phonologically realized noun phrase (Kornfilt, 1984; Özsoy, 1987). As a pro-drop language, Turkish may have clauses without overt subjects and the discourse-pragmatic context determines the choice between overt and null subjects (Enç, 1986; Taylan, 1986; Kerslake, 1987; Özsoy, 1987; Turan, 1995). Speakers of Turkish usually maintain referents with a null subject as in (b) taken from Azar et al. (2020):

(a) *Murat dün sinema-ya git-ti*.
  Murat yesterday cinema-DAT go-PAST.3SG
  “Murat went to the cinema yesterday.”
(b) *Ø film-i beğen-me-miş*.
  Ø movie-ACC like-NEG-PAST.EV.3SG
  “(He) did not like the movie.”

The example in (b) shows that the empty pronominal (Ø) is the counterpart of the overt subject pronoun (he) and its referent is determined by the subject-verb agreement (SVA) marking. If an overt subject pronoun or NP were used in example (b), this would not affect the truth value of the sentence because both versions (with null subject and overt subject pronoun or NP) of example (b) carry the same truth value. This illustrates that as long as the referent can be recovered from the context, the speaker may use a null subject in the sentence.

The 3rd person pronouns in Turkish (singular: *o* → he/she/it; plural *onlar* → they) do not encode gender or animacy (Azar et al., 2020). When a null subject is used for a 3rd person plural pronoun (e.g., as for *çocuklar* “children” from example c), the verb must always be marked with the 3rd person plural marker (*−lar/ler*) to avoid any ambiguity on the subject referent.

(c) *Çocuk-lar okul-dan çık-tı-(lar)*
  Child-PL school-ABL leave-PAST-(3PL)
  “Children left the school.”
(d) *Ø ev-e git-ti-ler*.
  Ø home-DAT go-PAST-3PL
  “(They) went home.”
(e) *Ø ev-e git-ti*.
  Ø home-DAT go-PAST.3SG
  “(He/She/It) went home.”

The empty pronominal in example (d) refers to the 3rd person plural pronoun *çocuklar* “children” in (c) because the verb is marked with the 3rd person plural marker. In contrast, the empty pronominal in example (e) does not refer to the 3rd person plural pronoun *çocuklar* “children” in (c) because the verb is not marked correctly, leading the subject referent to be infelicitous. While the correct interpretation of the empty pronominal must be *they* with the verb being marked correctly as in example (d), when the verb is not marked with the 3rd person plural suffix, the empty pronominal would be interpreted as *he/she/it* as in example (e).

On the other hand, when a referent is pragmatically marked for emphasis, contrast or topic shift, the overt subject is usually preferred over the null subject (Enç, 1986), as in (g) where the subject referent is marked for contrast and is expressed with an overt subject pronoun, *o* “she” instead of a null subject, which is taken from Azar et al. (2020):

(f) *Aynı film-i Suzan da izle-miş*.
  same movie-ACC Suzan too watch-PAST.EV.3SG
  “Suzan also watched the same movie.”
(g) *Ama o çok beğen-miş*.
  but she a lot like-PAST.EV.3SG
  “But she liked it a lot.”

### Previous studies in Turkish

The initial analyses on the use of overt and null subjects, which mainly focused on the overt and null pronouns in Turkish, were either theoretical (Enç, 1986; Taylan, 1986; Özsoy, 1987) or collected data from fiction novels (Kerslake, 1987; Turan, 1995). A few studies investigated the acquisition of null subjects in monolingual Turkish children. Slobin and Talay (1986) examined the speech transcripts of nine children between the ages of 24 to 56 months. They claimed that the subject in Turkish can be encoded by SVA marking alone or by an overt subject. Based on the analyses of all child utterances, they proposed that by the age of 24 months, SVA is correctly marked on verbs across a range of tenses, and both overt and null subjects are used by the children. They also found that both the morphosyntactic (SVA marking) and pragmatic (overt vs. null subjects) knowledge for marking the subject of a sentence are well established at early ages. Altan (2006, 2013) explored the use of null subjects in Turkish children grouped into three different age groups: age 2, age 3 and age 4 and replicated the results of Slobin and Talay (1986). She also observed that when children produce null subject sentences with 3rd person plural pronouns, they are adding the 3rd person plural marker on the verb correctly. Example (i) shows how a 2,8-year-old child participant marked the verb correctly although he was not expected to drop the subject pronoun because the experimenter was specifically asking about the subject.

(h) *O-nu kim al-dı san-a?*
  that-ACC who buy-PAST.3SG you-DAT.
  “Who bought that for you?”
(i) *Ø al-dı-lar.*
  Ø buy-PAST-3PL
  “(They) bought.”

There are also studies that focused on the use of null subjects in bilingual Turkish-speaking children who also speak a non-pro-drop language. Some of these studies found that Turkish children in contact situations are similar to monolingual Turkish children because they not only use the overt and null subjects to the same extent (Verhoeven, 1990; Aarssen, 1996) but also benefit effectively from the pragmatic context that requires the use of overt subjects (Özcan et al., 2000). Conversely, there are also studies that revealed differences between monolingual and bilingual Turkish-speaking children. In one of those studies, Haznedar (2010) collected spontaneous Turkish data from one Turkish-English bilingual child and one Turkish monolingual child. The results of the data comparison revealed that the Turkish-English bilingual child’s production of overt subjects in Turkish is more than the control child. This finding could be interpreted as due to cross-linguistic influence from English regarding the suppliance of overt subjects in the context of Turkish-English bilingual acquisition. In another study, Sağın Şimşek (2010) compared the use of overt and null subjects in four Turkish-German bilingual and four Turkish monolingual children who were between the ages of four to eight. The researcher found that the bilingual children use overt subjects more than the monolingual controls and attributed this result to the influence of German, which is a non-pro-drop language.

There are also studies that explore the use of overt and null subjects in adult non-heritage Turkish speakers. For example, Azar and Özyürek (2015) used two silent videos to elicit narration from non-heritage Turkish speakers (non-HS). The only personal pronoun the researchers observed in the experiment was 3^rd^ person singular pronoun (*o* → he/she/it) and they found that Turkish non-HS prefer overt subjects significantly more than null subjects to reintroduce subject referents. However, in the maintenance context, non-HS used null subjects significantly more. Similar findings were obtained in Azar et al. (2016, 2017) that used the same methodology. In another narration elicitation study, Azar et al. (2019) also observed sentences with 3rd person plural pronoun (*onlar* → they) and found that when the 3rd person plural pronoun is reintroduced, they are reintroduced with a null subject and the verb is marked with 3rd person plural marker to avoid any ambiguities regarding the subject referent as in example (k).

(j) *Üç tane bayan* var.
  three piece woman exist
  “There are three women.”
(k) *Ø yemek yap-ıyor-lar.*
  Ø food cook-PRES.CONT-3PL
  “(They) are cooking.”

As a result of the studies with adult non-heritage Turkish speakers, the researchers concluded that the use of subject in Turkish is primarily limited by pragmatic purposes; that is, overt subjects to mark emphasis and null subjects in the context of maintenance. These results clearly indicate that Turkish is a pro-drop language and in line with previous research in other pro-drop languages, null subject is the default form to maintain reference (e.g., Carminati, 2002).

Research has also been conducted with Turkish HS to examine the contact between pro-drop Turkish and a non-pro-drop language such as Dutch or English. Doğruöz (2007) analyzed the spoken corpora of Turkish in the Netherlands and Turkish in Turkey and found no effect of contact between the languages when the quantity of the subject pronouns in informal interviews were compared. Doğruöz and Backus (2009) analyzed the use of subject pronouns in informal interviews with Turkish HS living in the Netherlands and could not find any cross-linguistic effects when the amount of overt subjects is taken into consideration. They only observed a 2% of redundant overt subject use in the heritage data. In another study, Koban Koç (2016) interviewed HS living in New York City and the results showed that HS use overt subjects significantly higher than the non-HS living in Turkey. By using narrative elicitation of two silent videos, Azar et al. (2017, 2020) concluded that Turkish HS living in the Netherlands were similar to non-HS because HS perform similar to non-HS in terms of the discourse status or pragmatic constraints in the use of pronouns during speech. However, they also found a difference between the groups because HS use overt subjects slightly more than their non-HS peers. In addition, they observed that the reintroduction of a 3rd person plural pronoun is done with a null subject and the verb being correctly marked (example m).

(l) *İki kız masa-da sebze doğru-yor.*
  two girl table-LOC vegetable slice-PRES.CONT.3SG
  “Two girls are slicing vegetables on the table.”
(m) *Ø bir kavanoz aç-ma-ya çalış-ıyor-lar.*
  Ø a jar open-VN-DAT try-PRES.CONT-3PL
  “(They) are trying to open a jar.”

As can be seen, previous studies in Turkish have employed offline methods such as spoken corpora analysis, spontaneous speech, narrative elicitation and informal interviews and these studies have mainly focused on the conditions of the contextual and discourse/pragmatic appropriateness on the use of overt and null subjects because these conditions (i.e., external interface) regulate the choice of overt vs. null subjects in all pro-drop languages including Turkish. The HS studies have also investigated the contextual and discourse/pragmatic appropriateness *via* offline methods. Overall, the results are inconclusive and do not provide further evidence for the vulnerability/difficulty of external interfaces observed in HS. However, none of these studies have focused on the use of optional SVA marking (i.e., internal interface) with 3rd person plural subjects in sentences with overt subject NPs and null subjects. Previous research has shown that HS have difficulties with SVA marking (Benmamoun et al., 2013; Polinsky, 2018) making this phenomena of Turkish, that displays optionality, an interesting testing ground.

### Optional SVA marking in Turkish

Another important aspect in the use of null subjects is the SVA marking. Pro-drop languages typically display a rich inflectional morphology which allows for subjects to be dropped because agreement governs the empty *pro* category and helps to recover the dropped subjects (Cherici, 2021). Turkish verbs agree with the subjects in person and number (Enç, 1986) and Turkish marks subject agreement on the verbal element by means of a person suffix (Taylan, 1986). Like most agreement-marking languages, singular subjects require singular verb forms and plural subjects require plural verb forms. However, Turkish SVA marking is an example of optional agreement in which the 3rd person plural subject has optionality and can be used with singular (unmarked) verb forms under certain conditions. Example (n) illustrates this optionality, where the omission of plural suffix *-lar/ler* renders the verb form indistinguishable from the 3rd person singular form.

(n) *Öğrenci-ler okul-dan gel-di-(ler)*.
  student-PL school-ABL come-PAST-(3PL)
  “Students came from school.”

Turkish non-HS usually have a tendency to avoid using the same morphological marker within the same clause or phrase. According to Johanson (1998), this is a general tendency in Turkic languages with an attempt to use morphological devices economically and avoid redundancy. Previous research with Turkish non-HS has shown that for sentences with 3rd person plural subjects, the acceptability of a plural marker on the verb is affected by semantic and pragmatic factors such as the subject’s degree of animacy (e.g., Schroeder, 1999; Bamyacı et al., 2014). While animate plural subjects may take either a plural or an unmarked verb (example o), inanimate plural subjects usually take an unmarked verb (example p) (Sezer, 1978). Using plural marked or unmarked verb forms with animate plural subjects depends on the speaker’s stylistic preferences without a difference regarding the meaning (Sezer, 1978; Kornfilt, 1997).

(o) *Çilingir-ler kapı-lar-ı aç-tı-(lar)*.
  locksmith-PL door-PL-ACC open-PAST-(3PL)
  “Locksmiths opened the door.”
(p) Anahtar-lar kapı-lar-ı aç-tı-(*lar)
  Key-PL door-PL-ACC open-PAST-(*3PL)
  “Keys opened the door.”

As for the HS, several acceptability judgment studies have been conducted. For example, Bamyacı (2016) found that they are similar to non-HS in the way they treat SVA with 3rd person plural subjects, but she also observed that HS have a greater likelihood of accepting plural-marked verbs. Lago et al. (2019) found that while non-HS prefer unmarked verb forms with animate 3rd person plural subjects, HS accept both plural-marked and unmarked forms to a similar extent. Recently, Uygun and Felser (2021) reported that HS rate plural verb forms better when the subject was animate, but did not find a difference in the overall acceptance of plural-marked vs. unmarked verbs between HS and non-HS.

To summarize, previous studies on the optional SVA marking with HS have displayed several differences when compared to the non-HS. These results are in line with the predictions that phenomena displaying optionality are more affected under HL conditions resulting in differences when compared to non-HS (Benmamoun et al., 2013). All of the above-mentioned studies provide us information about the metalinguistic judgment of the HS when there was no time constraint, yet it is not known how they would perform in sentences with overt subject NPs and null subjects when their reaction times are measured.

### The study

Since most existing studies that have compared overt and null subjects have tested the use of overt pronouns, the main purpose of the current study was to investigate the reading times (RTs) of plural-marked and unmarked verbs in sentences with overt subject NPs and null subjects during real time sentence processing. While previous studies have always focused on the contextual and discourse/pragmatic appropriateness of using overt vs. null subjects (i.e., external interface), this is the first study to explore the optional SVA marking (i.e., internal interface) in a pro-drop language. While the verb can be either plural-marked or unmarked in sentences with 3rd person animate plural subjects, the verb must be always plural-marked for sentences when a null pronoun replaces the 3rd person animate plural subject. By employing a self-paced reading experiment, it was expected to gain more insights into implicit processing preferences for the optional SVA marking in sentences with both overt subject NPs and null subjects and make a comparison between heritage and non-heritage speakers of Turkish. Since offline tasks do not offer direct access to participants’ mental processes as they unfold in real time, it was decided to use an online task, which measures participants’ automatic responses to language stimuli, providing a more direct access to *how* language processing unfolds in real time (Bayram et al., 2021). In a self-paced reading task, participants read sentences presented to them one word or phrase at a time on the computer screen. According to Bayram et al. (2021), the main goal of this task is not to make a quantitative comparison by focusing on the reading times of HS and non-HS group but to understand whether HS process their HL qualitatively different from non-HS of the same language.

The following research questions were sought to investigate:

1. Is there a difference between Turkish HS and non-HS in their RTs of plural marked and unmarked verbs in sentences with overt subject NPs and null subjects?
2. Is the optional SVA marking in Turkish, which is considered as an internal interface, vulnerable/problematic to acquire and cause a processing load for HS?

Based on the previous results, non-HS are expected to show no RT differences for plural marked vs. unmarked verbs in sentences with overt subject NPs because they have no problems with the optional SVA marking in Turkish. Conversely, for null subject sentences, non-HS are expected to display longer RTs for unmarked verbs because unmarked verbs cause a mismatch between the subject and the verb. As for the HS, they are expected to show shorter RTs for plural marked verbs in sentences with overt subject NPs which is indicative of their problems with the optional agreement marking, providing evidence for their struggle with operations that involve computation across more than one grammatical component (e.g., syntax and morphology) and that internal interfaces are also vulnerable for them (Benmamoun et al., 2013). In addition, they are expected to perform similar to the non-HS in sentences with null subjects as SVA marking is compulsory. If no difference is observed between HS and non-HS in sentences with overt subject NPs and null subjects, this would support the revised version of the IH (Sorace and Serratrice, 2009) by showing that internal interfaces are not difficult to acquire and process.

## Materials and methods

### Participants

Forty non-heritage Turkish speakers (non-HS) were recruited and tested in Istanbul. All non-HS participants were born and raised in Turkey and they had never lived abroad. One non-HS participant was excluded due to high error rates in the filler condition (> 30%). The remaining 39 non-HS participants (mean age = 36.87, SD = 9.21, age range = 19–60, 29 females) were either university graduates or were studying at the university at the time of testing and they all spoke the standard dialect of Turkish. 60 Turkish heritage speakers (HS) residing in Berlin and Potsdam were recruited and tested. All of the HS in the study were exposed to Turkish from birth and spoke both Turkish and German in their daily lives. One participant was excluded due to low Turkish proficiency score (below 12 out of 20 indicating a proficiency level lower than B2 level) from the Turkish TELC (The European Language Certificates) test which is designed for B2 level based on the Common European Framework of Reference (CEFR). The language structure part of the TELC test consists of two cloze tests with 20 questions in total. The remaining data of 59 HS (mean age = 27.78, SD = 6.06, age range = 19–50, 42 females) were analyzed. All HS had an early age of acquisition of German (mean age = 3.01, SD = 1.85, age range = 0–6) and a high score from the Turkish TELC (mean score = 18.44, SD = 1.62, score range = 13–20). In addition, the HS group self-rated their weekly use of Turkish and the results show a predominant use of Turkish in a normal week covering reading, writing, speaking, and listening, with a mean rate of 61.61% (SD = 21.82, range = 15–90). The HS group also self-rated their Turkish proficiency level out of 10 across four language skills and the results revealed a high proficiency level for the HS (Speaking: mean = 7.91, SD = 1.63; Listening: mean = 8.84, SD = 1.17; Writing: mean = 7.17, SD = 2.04; Reading: mean = 8.21, SD = 1.70). Both the TELC scores and the self-ratings indicate a high level of Turkish proficiency in the HS. All participants received a small fee for their participation.

### Materials

The present experiment had a factorial design with two within-participant factors and group as the between-participant factor. By manipulating the existence of the subject (null subject vs. overt subject NP) and verb marking (plural-marked vs. unmarked), 24 experimental sentence sets were created with four different conditions as illustrated in (q-t). All experimental sentences had a context sentence and the subject was always a 3rd person plural animate subject to investigate the optional SVA marking. For null subject sentences, the context sentence is in the SOV order and the verb is always unmarked. The context sentences in the present experiment do not aim to explore the contextual and discourse/pragmatic appropriateness on the use of overt and null subjects. The context sentence below for examples (q) and (r) actually facilitates the use of null subjects. The NS-PL condition in example (q) is correct because the subject in the context sentence *polisler* “policemen” is a 3rd person plural animate subject and the verb in example (q) has the plural marker *–ler* in the end (*götürdüler* “took”) and therefore the subject referent is unambiguous. In contrast, in the NS-SG condition in example (r), the verb is unmarked (*götürdü* “took”) causing a mismatch between the subject referent and the verb and making the subject referent infelicitous.

Context sentence for null subject sentences:
*Polis-ler dün genç hırsız-ı yakala-dı*.
police-PL yesterday young thief-ACC catch-PAST.3SG.
“The policemen caught the young thief yesterday.”

(q) Null subject – Plural (NS-PL):
*Hırsız-ı karakol-a götür-dü-ler ama hırsız kaç-tı.*
  thief-ACC police station-DAT take-PAST-3PL but thief run away-PAST.3SG
  “(The policemen) took the thief to the police station, but the thief ran away.”
(r) Null subject – Singular (NS-SG):
*Hırsız-ı karakol-a götür-dü ama hırsız kaç-tı.*
  thief-ACC police station-DAT take-PAST.3SG but thief run away-PAST.3SG
  “(The policemen) took the thief to the police station, but the thief ran away.”

The same context sentence for overt subject NP sentences is transformed into the passive voice without providing the agent, which facilitates the use of an overt subject NP for sentences in examples (s) and (t). In both OS-PL and OS-SG conditions, the subject is a 3rd person plural animate subject (*polisler* “policemen”). This means that the verb can be used as both plural-marked as in example (s) (*götürdüler* “took”) or unmarked as in example (t) (*götürdü* “took”). Both versions are grammatically correct and they do not differ in terms of meaning.

Context sentence for overt subject sentences:
*Dün genç hırsız yakala-n-dı*.
yesterday young thief catch-PSV-PAST.3SG.
“The young thief was caught yesterday.”

(s) Overt subject – Plural (OS-PL):
*Polis-ler hırsız-ı karakol-a götür-dü-ler ama hırsız kaç-tı.*
  police-PL thief-ACC police station-DAT take-PAST-3PL but thief run away-PAST.3SG
  “The policemen took the thief to the police station, but the thief ran away.”
(t) Overt subject – Singular (OS-SG):
*Polis-ler hırsız-ı karakol-a götür-dü ama hırsız kaç-tı.*
  police-PL thief-ACC police station-DAT take-PAST.3SG but thief run away-PAST.3SG
  “The policemen took the thief to the police station, but the thief ran away.”

Four different presentation lists were created in a Latin-square design and the items in each version were pseudo-randomized and mixed with 48 filler sentences, resulting in a total of 72 items per list.

### Design and procedure

The experiment was designed on the web-based platform Ibex Farm (Drummond, 2013) and the sentences were presented word-by-word using the noncumulative moving window paradigm (Just et al., 1982). Each trial began with a screen presenting a sentence in which the words were masked by underscores. When the participant pressed the space bar button, a word was revealed and the previous word was re-masked. After reaching the final word of the sentence which appeared with a full stop, the participants pressed the space bar button again to decide if the second sentence was a grammatically and semantically good continuation of the context sentence by pressing “f” for “yes” and “j” for “no” response. After their response, they had to press the space bar button for the next trial.

The experiment began by requesting participants to complete a demographic questionnaire and give their consent. Then, they were instructed to read the sentences carefully and answer the questions as quickly as possible. Five practice items were presented to familiarize the participants with the procedure. Participants received a link to the experiment and completed the test on their personal computers. A progress bar shown above the sentences allowed them to keep track of their progress. The experiment took approximately 20 min, and the HS group additionally completed the Turkish proficiency test afterwards.

## Results

The dependent measures were word-by-word RTs of different regions in the experimental sentences. The main interest was in obtaining significant group differences in these regions. For word-by-word reading data, RTs exceeding 2.5 standard deviations above and below a participant’s mean log reading time were deemed outliers and removed (HS group = 2.76%; non-HS group = 3.11%). To counter the problems of word length and individual differences in reading times, residual reading times (RRTs) were calculated on the remaining data with linear modelling on the log transformed RTs. Positive values indicate that a reading time is slower than expected whereas negative values indicate a faster reading time. RRTs were analysed for five regions of interest: the critical region of “The verb,” the pre-critical region of “Before the verb” and the three “Spillover” regions following the critical region (see Table 1 below for the regions, analyses were conducted starting from Region 3).

**Table 1:** Regions of interest in the experimental sentences.

Regions	Null subject	Overt subject	Example
Region 1	*Not applicable*	Subject	Polisler (*The policemen*)
Region 2	Object	Object	hırsızı (*the thief*)
Region 3	Before the verb	Before the verb	karakola (*to the police station*)
Region 4	The verb	The verb	götürdü(ler) (*took-SG or PL*)
Region 5	Spillover 1	Spillover 1	ama (*but*)
Region 6	Spillover 2	Spillover 2	hırsız (*the thief*)
Region 7	Spillover 3	Spillover 3	kaçtı (*ran away*)

Statistical analyses were conducted with R, an open-source programming language and environment for statistical computing (R Development Core Team, 2017). The RRTs data were analyzed with linear mixed-effects regression models with crossed random effects for items and subjects (Baayen et al., 2008). The models were fitted using the package *lme4* (Bates et al., 2015). The models included the subject-level variable “Group” (HS vs. non-HS) and the item-level variables “The Existence of Subject” (null subject vs. overt subject NP) and “Verb Marking” (plural marked vs. unmarked) as fixed effects. The model also included random intercepts for item and subject. For the main effects and overall interactions, sum-coded contrasts (−0.5, 0.5) were employed to the factors Group, The Existence of the Subject and Verb Marking. For single comparisons, treatment contrasts were applied. Initially, a model with random intercepts and slopes for all fixed effects and their interactions was constructed and when this maximal model failed to converge, it was gradually simplified until convergence was reached (Barr et al., 2013). The Akaike Information Criterion (AIC) was used for model comparison because it provides a measure that penalizes complexity and leads to predictors being kept only when they substantially contribute to explaining variance in the data (Venables and Ripley, 2002). The model with the lower AIC value was selected and this procedure was repeated until the simplification process did not produce a model with a lower AIC. The final version of the model included by item and by subject random slopes for the interaction of the existence of the subject and verb marking. The effect sizes are reported by using model coefficients in log odds (*ß*), standard errors (*SE*), *t*-statistics and *p* values. *P*-values were computed by using the *lmerTest* package and the Satterthwaite’s approximation for denominator degrees of freedom (Kuznetsova et al., 2014).

**Figure 1:**
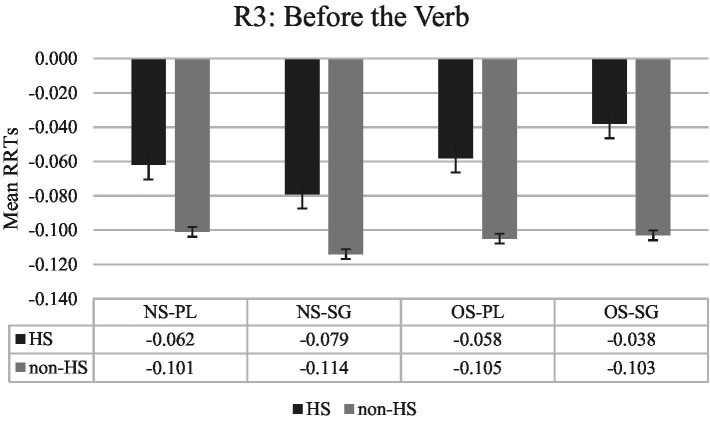
Mean RRTs of both groups for Region 3 (Before the verb). RRTs, Residual reading times; HS, Heritage speakers; non-HS, Non-heritage speakers; NS, Null subject; OS, Overt subject; PL, Verb is plural-marked; SG, Verb is unmarked.

The first region of interest in the experiment is region 3 (Before the verb), the region prior to the critical (see Figure 1) region. The RRTs analysis of this region indicate a significant main effect of group only (see Table 2). The non-HS group had significantly faster RRTs than the HS group (*ß*: 0.046, *SE:* 0.016, *t* = 2.958, *p* < 0.005) in the pre-critical region “Before the verb.”

**Table 2:** Linear mixed effects model output for Region 3 (Before the Verb).

	*ß*	*SE*	*t*	*P*
Intercept	−0.082	0.011	−7.226	0.000*
Subject (Null subject vs. Overt subject)	−0.014	0.012	−1.223	0.222
Verb marking (Plural-marked vs. Unmarked)	0.001	0.013	0.091	0.928
Group (HS vs. non-HS)	0.046	0.016	2.958	0.004*
Subject*Verb marking	0.026	0.029	0.898	0.370
Subject*Group	−0.016	0.023	−0.725	0.468
Verb marking*Group	−0.005	0.025	−0.190	0.850
Subject*Verb marking*Group	0.022	0.048	0.457	0.648

Formula in R: RTresidual ~ Subject*Verb marking*Group + (1 + Subject*Verb marking | item) + (1 + Subject*Verb marking | subject). Bold values indicate the significant results.

**Figure 2:**
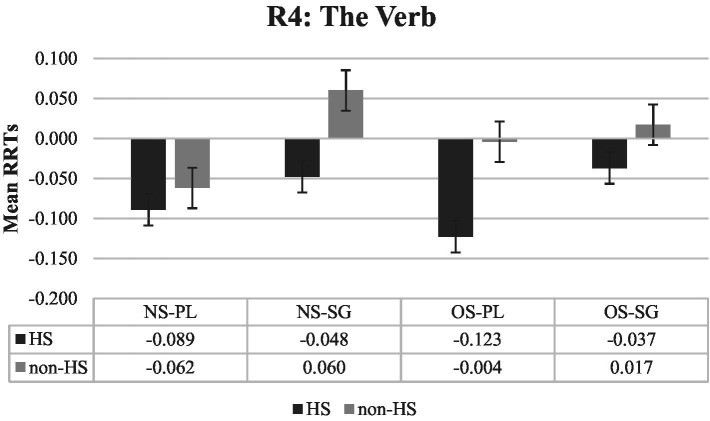
Mean RRTs of both groups for Region 4 (The verb). RRTs, Residual reading times; HS, Heritage speakers; non-HS, Non-heritage speakers; NS, Null subject; OS, Overt subject; PL, Verb is plural-marked; SG, Verb is unmarked.

The second region of interest is region 4 (The verb), which is the critical region in the experiment (see Figure 2). In this region, significant main effects of verb marking (*ß*: −0.068, *SE:* 0.020, *t* = −3.368, *p* < 0.001) and group (*ß*: −0.079, *SE:* 0.023, *t* = −3.510, *p* < 0.001) and a significant three-way interaction of the existence of subject, verb marking, and group (*ß*: 0.140, *SE:* 0.055, *t* = 2.528, *p* < 0.013) were obtained (see Table 3). The effect of verb marking indicates that plural-marked verbs receive significantly shorter RTs than the unmarked verbs and HS had significantly faster RTs than the non-HS group. To resolve the significant existence of subject, verb marking and group interaction, each group was analyzed separately. For the non-HS group, a significant main effect of verb marking (*ß*: −0.072, *SE:* 0.032, *t* = −2.243, *p* < 0.027) and a significant interaction between the existence of subject and verb marking (*ß*: 0.097, *SE:* 0.048, *t* = 2.009, *p* < 0.045) were obtained. Plural-marked verbs receive significantly shorter RTs than unmarked verbs in general. The significant interaction reveals that in null subject sentences, unmarked verbs take significantly longer to read than plural-marked verbs (*ß*: −0.119, *SE:* 0.044, *t* = −2.753, *p* < 0.007); however, in overt subject NP sentences, unmarked verbs take numerically longer to read (*ß*: −0.022, *SE:* 0.039, *t* = −0.562, *p* < 0.575). For the HS group, there was only a significant main effect of verb marking (*ß*: −0.064, *SE:* 0.021, *t* = −3.091, *p* < 0.003) indicating that plural-marked verbs receive significantly shorter RTs than unmarked verbs.

**Table 3:** Linear mixed effects model output for Region 4 (The Verb).

	*ß*	*SE*	*t*	*P*
Intercept	−0.033	0.019	−1.756	0.080
Subject (Null subject vs. Overt subject)	0.004	0.015	0.265	0.792
Verb marking (Plural-marked vs. Unmarked)	−0.068	0.020	−3.368	0.000*
Group (HS vs. non-HS)	−0.079	0.023	−3.510	0.000*
Subject*Verb marking	−0.026	0.035	−0.729	0.466
Subject*Group	0.017	0.028	0.628	0.530
Verb marking*Group	0.008	0.030	0.281	0.778
Subject*Verb marking*Group	0.140	0.055	2.528	0.012*

Formula in R: RTresidual ~ Subject*Verb marking*Group + (1 + Subject*Verb marking | item) + (1 + Subject*Verb marking | subject).

**Figure 3:**
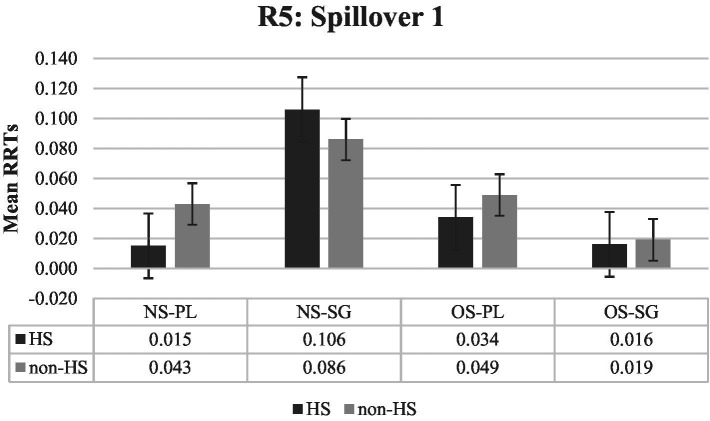
Mean RRTs of both groups for Region 5 (Spillover 1). RRTs, Residual reading times; HS, Heritage speakers; non-HS, Non-heritage speakers; NS, Null subject; OS, Overt subject; PL, Verb is plural-marked; SG, Verb is unmarked.

Figure 3 illustrates the RRTs for region 5 (Spillover 1), which comes right after the critical region. A significant main effect of the existence of subject (*ß*: 0.033, *SE:* 0.015, *t* = 2.197, *p* < 0.029) and a significant interaction between the existence of subject and verb marking (*ß*: −0.091, *SE:* 0.032, *t* = −2.864, *p* < 0.005) were obtained in this region (see Table 4). In general, null subject sentences receive significantly longer RTs than overt subject NP sentences. Regarding the significant interaction, in null subject sentences, unmarked verbs take significantly longer than plural-marked verbs (*ß*: −0.068, *SE:* 0.022, *t* = −3.109, *p* < 0.003) while in overt subject NP sentences, plural-marked verbs take numerically longer to read (*ß*: 0.023, *SE:* 0.019, *t* = 1.215, *p* < 0.225). In addition, when the verb was unmarked, null subject sentences take significantly longer to read (*ß*: 0.079, *SE:* 0.023, *t* = 3.427, *p* < 0.001). However, there is no significant difference when the verb was plural-marked (*ß*: −0.012, *SE:* 0.017, *t* = −0.723, *p* < 0.471).

**Table 4:** Linear mixed effects model output for Region 5 (Spillover 1).

	*ß*	*SE*	*t*	*P*
Intercept	0.046	0.007	6.255	0.000*
Subject (Null subject vs. Overt subject)	0.033	0.015	2.197	0.028*
Verb marking (Plural-marked vs. Unmarked)	−0.022	0.016	−1.367	0.172
Group (HS vs. non-HS)	−0.006	0.012	−0.537	0.592
Subject*Verb marking	−0.091	0.032	−2.864	0.004*
Subject*Group	0.005	0.027	0.186	0.852
Verb marking*Group	−0.027	0.027	−1.014	0.310
Subject*Verb marking*Group	−0.039	0.057	−0.677	0.498

Formula in R: RTresidual ~ Subject*Verb marking*Group + (1 + Subject*Verb marking | item) + (1 + Subject*Verb marking | subject). Bold values indicate the significant results.

**Figure 4:**
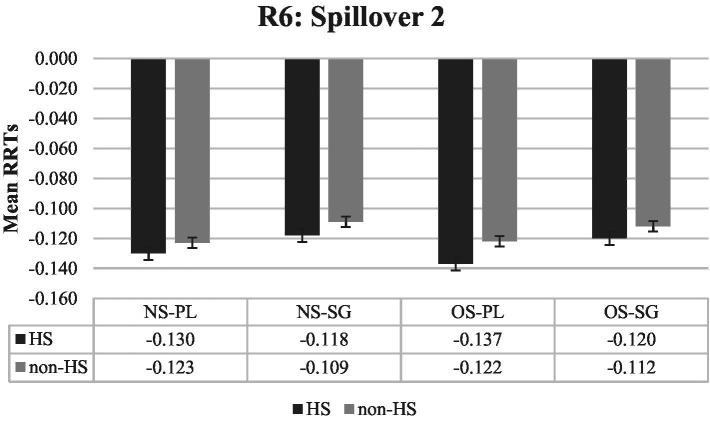
Mean RRTs of both groups for Region 6 (Spillover 2). RRTs, Residual reading times; HS, Heritage speakers; non-HS, Non-heritage speakers; NS, Null subject; OS, Overt subject; PL, Verb is plural-marked; SG, Verb is unmarked.

**Figure 5:**
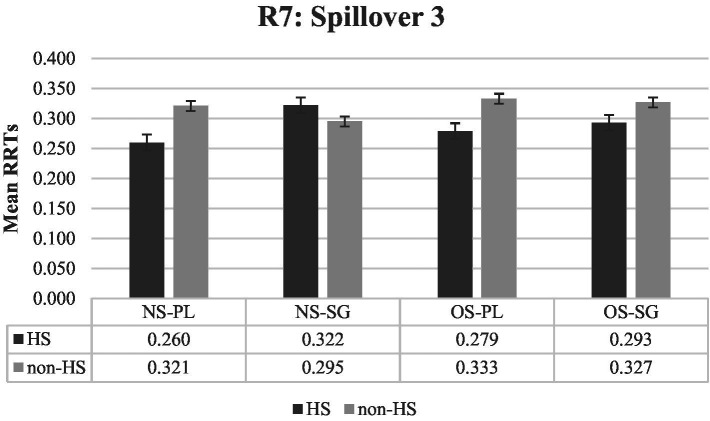
Mean RRTs of both groups for Region 7 (Spillover 3). RRTs, Residual reading times; HS, Heritage speakers; non-HS, Non-heritage speakers; NS, Null subject; OS, Overt subject; PL, Verb is plural-marked; SG, Verb is unmarked.

Finally, as illustrated by Figure 4 and 5, there were no significant main effects or interactions in the last two regions, namely region 6 and 7 (Spillover 2 and 3).

## Discussion

By carrying out an online experiment that measures the RTs, the present study tried to explore the RRTs for plural-marked and unmarked verbs in order to investigate the optional SVA marking with heritage and non-heritage speakers of Turkish when they read sentences with overt subject NPs and null subjects.

The results suggest both quantitative and qualitative differences between the HS and non-HS groups in the critical region “The verb.” First of all, HS are significantly faster than non-HS in this region. Secondly, the HS group has significantly faster RTs for the plural marked verbs in general, indicating that their RTs are not affected by the manipulation of the existence of the subject. This also means that HS prefer plural-marked verbs both in overt subject NP and null subject sentences. While this tendency is correct for the null subject sentences to prevent the subject referent ambiguity, it clearly shows their difficulty in using the optional SVA marking in overt subject NP sentences. Conversely, the non-HS group displays a significant interaction of the existence of subject and verb marking in this region. Similar to the HS group, non-HS favor plural-marked verbs in sentences with null subject to keep the subject referent unambiguous. However, for sentences with overt subject NP, the non-HS group behaves differently from the HS group because there is no significant RT difference between plural-marked and unmarked verbs, which indicates the use of optional SVA marking with no difficulty.

Yet, in the “Spillover 1” region, which comes right after the critical region “The verb,” both groups behave similarly and there are not any quantitative or qualitative differences. First of all, there is no significant RT difference between the groups. Secondly, in this region, the existence of the subject affects both groups in the same way with significantly longer RTs for sentences with null subject. In addition, the significant interaction of the existence of subject and verb marking reveals that unmarked verbs take significantly longer to read than plural-marked verbs in null subject sentences because they cause a mismatch between the subject and the verb, but there is no significant difference between plural-marked and unmarked verbs in overt subject NP sentences indicating no difficulty for the optional SVA marking.

How can the observed between-group difference in the critical region “The verb” be accounted for? Recall that the IH makes a clear distinction between internal and external interfaces for bilinguals including HS. While internal interfaces involve interactions between language modules (e.g., syntax and morphology), external interfaces have interactions between linguistic and non-linguistic cognitive systems (e.g., syntax and discourse). The IH predicts processing limitations to be affected only in external interfaces because structures that require internal mappings are less taxing than structures that require external mappings (Sorace, 2011, 2012). In contrast to this view, Benmamoun et al. (2013) claims that HS experience problems when they have to compute interface properties without making a distinction between internal and external interfaces. There are studies that found evidence to support the claims of Benmamoun et al. (2013). For example, Benmamoun (2000) investigated the construct state used to form genitive construction in Arabic and observed that HS do not treat the construct state as a single prosodic unit because they were using double marking. This divergence indicated that HS fail to compute the internal interface between syntax and phonology. Internal interface effects have also been observed by Albirini et al. (2011) in Arabic agreement and coordination, which relies on the interaction between syntax and the morpho-phonological component of the grammar. The authors suggested that HS could no longer control the interface between syntax and the morpho-phonology in their grammars. In another study, Mendez et al. (2015) found that HS perform differently from non-HS with internal interface properties as well, claiming that internal interface properties are also difficult to acquire. In addition, Gondra (2022) concluded that HS are vulnerable to both internal (syntactic-semantic) and external (pragmatic-discursive) interfaces. Another example of the internal interface is the optional SVA marking, which the current study investigates. According to Benmamoun et al. (2013), HS are expected to have difficulty with interfaces between syntax and morphology and these interfaces are predicted to be more difficult to acquire or more vulnerable to attrite. The data in the critical region “The verb” shows that HS have difficulties with the internal interface of syntax (sentences with overt subject NPs vs. null subjects) and morphology (plural-marked vs. unmarked verbs) because they behave differently from non-HS when they have to integrate these two language modules.

In addition, it is also known that controlling two languages has significant impacts on linguistic and general cognitive abilities leading to several advantages and disadvantages for bilinguals (Sorace, 2011). Because both languages are simultaneously activated in the bilingual mind even in cases when one is contextually unnecessary (Marian et al., 2003; Bialystok, 2009), bilingual processing is predicted to be less efficient than monolingual processing. Sorace (2011) claims that bilinguals are less efficient than monolinguals because their knowledge of or access to computational constraints within the language module is less detailed and/or less automatic than in monolinguals and because they have fewer general cognitive resources to deploy on the integration of different types of information in online language comprehension and production. According to Sorace (2011), accessing and integrating two types of knowledge is *more costly* than accessing only one type of knowledge and the problem mainly lies in the bilinguals’ less optimal ability to consistently and effectively integrate different types of knowledge. Since HS are considered as a subgroup of bilinguals, these claims are directly relevant to HS as well although this problem is expected to be smaller for HS in comparison to L2 speakers. The online integration of different types of knowledge may incur a cost for HS as they may be less efficient in integrating diverse knowledge when compared to the non-HS group. In the current experiment, the online integration of syntactic knowledge (whether the sentence has an overt subject NP vs. null subject) and morphological knowledge (whether the verb is plural-marked or unmarked) is a demanding task that requires a lot of cognitive demands (Rothman and Slabakova, 2011). Because HS fail to integrate these different types of knowledge successfully, they are found to be significantly different from the non-HS group in the critical region “The verb.” Polinsky and Scontras (2020) recently proposed that HS are likely to face difficulty with phenomena that impose cognitive demands as a result of their processing resource limitations. They claim that HS restructure their grammar to free up processing resources resulting in a change in their grammar. The limited nature of their processing resources in the non-dominant language forces their grammar to be less ambiguous, more regular and having less structure. For the optional SVA marking, “the restructuring of grammar” means that HS try to regularize the optional SVA system by over-using plural suffixes in contexts in which non-HS prefer to use the unmarked verb forms. This limitation may explain why the integration process of syntactic and morphological knowledge incurs a cost for the HS as they behave differently from the non-HS group only in the critical region “The verb” and prefer the plural-marked verbs more regardless of the existence of the subject.

Another important factor that may lead to difficulties in integrating different types of knowledge is the quality and quantity of input that HS receive. According to Polinsky and Scontras (2020), less time dedicated on a language leads to reduced input, which is considered to be a crucial factor that leads to the observed divergences between heritage and non-heritage speakers. Regarding the quantity of input, they claim that different grammatical phenomena might be sensitive to input quantity. For example, if a phenomenon is rare and not reinforced, HS will never encounter the necessary input to learn the phenomenon successfully. For the input quality, they assert that HS’ input is limited to a small set of speakers and the topics common to the situations in which the HL is used. Mendez et al. (2015) also suggest that any changes in the input quality of HS would result in displaying less sensitivity to appropriate grammatical choices, especially for structures that allow for two or more options which must be inferred from the reduced or suboptimal linguistic input conditions. In addition, researchers have recently agreed on the vital role of both the quality and quantity of input in integrating knowledge from different sources (Chondrogianni and Marinis, 2011; Kupisch et al., 2014; Unsworth et al., 2014; Unsworth, 2016). Therefore, it can be concluded that both the quantity and quality of input play a central role for the observed performance of HS in the present study.

What is more, from a methodological point of view, the online nature of the task provides additional information about the temporal resolution of the processing rather than the metalinguistic knowledge of the participant which is provided by judgment tasks. Online tasks can provide information about the point at which the integration of information from different sources becomes difficult and thus leading to different processing patterns (Sorace, 2011). This is exactly what was observed in the present study. While the HS performed differently from the non-HS group in the critical region “The verb,” they behaved similar to the non-HS group in the next region. This shows that HS face difficulties in integrating the syntactic and morphological knowledge in the critical region “The verb” and they need more time to integrate this knowledge compared to the non-HS. Yet, after this critical point, their processing mechanism functions in the same way as the non-HS group as no group differences are observed in the “Spillover” regions. Previous studies with HS on the optional SVA marking in Turkish have used the acceptability judgment task, which is an offline task that mainly measures the metalinguistic knowledge. While two studies have reported an over-acceptance of plural-marked verbs among HS (Bamyacı, 2016; Lago et al., 2019), one study has revealed no difference in the overall acceptance of plural-marked vs. unmarked verbs between HS and non-HS (Uygun and Felser, 2021). The over-use of plural-marked verbs in both offline and online tasks may be attributed to the less robust grammar of HS. According to Putnam (2019), HS develop unstable and unconsolidated grammars as a result of the competition between their (two or) more languages when compared to non-HS.

These processing resource limitations that lead to the restructuring of grammar and the reduced input conditions may explain why the integration process of knowledge from two different sources incurs a cost for the HS group as they display the existence of subject and verb marking interaction only in the “Spillover 1” region but not in the critical region “The verb” while the non-HS group shows this interaction both in “The verb” and “Spillover 1” regions. The HS group experiences problems when they have to integrate the syntactic and morphological knowledge (i.e., internal interfaces) as they are less affected by the existence of subject in the same way as the non-HS group. The non-HS group was able to contrast the two subject conditions more strongly than the HS in the critical region of ‘The verb’ while the HS group failed to contrast this manipulation. But more real-time processing research in optional SVA marking with HS is needed to assess and compare HS and non-HS groups’ linguistic behavior and performance to be able to reach more generalizable conclusions regarding internal interfaces.

## Conclusion

Since the acquisition of the phenomena displaying optionality is known to be difficult as a result of the suboptimal input and acquisition conditions, the optional SVA marking of Turkish has been investigated in HS and non-HS by employing a self-paced reading experiment that measures the reading times of the words. SVA marking is an internal interface involving the combination of syntactic and morphological knowledge and is not expected to be difficult to acquire and vulnerable to attrite. The results indicate that HS behave differently from non-HS even in internal interfaces and the nature of the experiment enables us to see at which point(s) there are quantitative and qualitative differences between the groups and whether HS restructure their grammar to compensate for their processing problems under time pressure. It is very important to test different phenomena in HS *via* online and offline measures with an attempt to understand the factors that make HS and their native language different from the non-HS and to obtain a comprehensive picture of theories about bilingualism and heritage language.

## Data availability statement

The raw data supporting the conclusions of this article will be made available by the author, without undue reservation.

## Ethics statement

The studies involving human participants were reviewed and approved by the University of Potsdam. The patients/participants provided their written informed consent to participate in this study.

## Author contributions

The author designed the experiment, analysed the data, wrote the manuscript and has approved it for publication.

## Funding

This research was funded by the Deutsche Forschungsgemeinschaft (DFG, German Research Foundation) – Project Number: 317633480 – SFB 1287 Project B04.

## Conflict of interest

The author declares that the research was conducted in the absence of any commercial or financial relationships that could be construed as a potential conflict of interest.

## Publisher’s note

All claims expressed in this article are solely those of the authors and do not necessarily represent those of their affiliated organizations, or those of the publisher, the editors and the reviewers. Any product that may be evaluated in this article, or claim that may be made by its manufacturer, is not guaranteed or endorsed by the publisher.
