# Supplementary material for: Processing pro-drop features in heritage Turkish
Source: Front Psychol. 2022 Nov 15;13:988550. doi: 10.3389/fpsyg.2022.988550 (PMC9707335; doi:10.3389/fpsyg.2022.988550)
Supplement: Supplementary file 1 [file Table_1.DOCX]

**Self-paced Reading Experimental Items**

24 set of experimental items are created.

For null subject (NS) sentences:

- Context sentences (CS) are created. They are SOV sentences with 3^rd^ person plural animate subject and the verb is always unmarked
- The first experimental sentence has an unmarked (SG) verb form
- The second experimental sentence has a plural-marked (PL) verb form

For overt subject (OS) sentences:

- The same context sentence (CS) is transformed into passive voice (without providing the agent), in the experimental sentences, a 3^rd^ person plural animate subject is provided
- The first experimental sentence has an unmarked (SG) verb form
- The second experimental sentence has a plural-marked (PL) verb form

1. CS-1: Polisler dün genç hırsızı yakaladı. *The policemen caught the young thief yesterday.*

NS-SG-1: Hırsızı karakola götürdü ama hırsız kaçtı. *(The policemen) took(SG) the thief to the police station but the thief ran away.*

NS-PL-1: Hırsızı karakola götürdüler ama hırsız kaçtı. *(The policemen) took(PL) the thief to the police station but the thief ran away.*

CS-1: Dün genç hırsız yakalandı. *The young thief was caught yesterday.*

OS-SG-1: Polisler hırsızı karakola götürdü ama hırsız kaçtı. *The policemen took(SG) the thief to the police station but the thief ran away.*

OS-PL-1: Polisler hırsızı karakola götürdüler ama hırsız kaçtı. *The policemen took(PL) the thief to the police station but the thief ran away.*

2. CS-2: Çocuklar bahçede bir kedi gördü. *The children saw a cat in the garden.*

NS-SG-2: Kediyi sınıfa getirdi ve kedi uyudu. *(The children) brought(SG) the cat to the classroom and the cat slept.*

NS-PL-2: Kediyi sınıfa getirdiler ve kedi uyudu. *(The children) brought(PL) the cat to the classroom and the cat slept.*

CS-2: Bahçede bir kedi görüldü. *A cat was seen in the garden.*

OS-SG-2: Çocuklar kediyi sınıfa getirdi ve kedi uyudu. *The children brought(SG) the cat to the classroom and the cat slept.*

OS-PL-2: Çocuklar kediyi sınıfa getirdiler ve kedi uyudu. *The children brought(PL) the cat to the classroom and the cat slept.*

3. CS-3: Öğretmenler okulda bir çanta buldu. *The teachers found a bag in the school.*

NS-SG-3: Çantayı bahçede açtı ama çanta boştu. *(The teachers) opened(SG) the bag in the garden but the bag was empty.*

NS-PL-3: Çantayı bahçede açtılar ama çanta boştu. *(The teachers) opened(PL) the bag in the garden but the bag was empty.*

CS-3: Okulda bir çanta bulundu. *A bag was found in the school.*

OS-SG-3: Öğretmenler çantayı bahçede açtı ama çanta boştu. *The teachers opened(SG) the bag in the garden but the bag was empty.*

OS-PL-3: Öğretmenler çantayı bahçede açtılar ama çanta boştu. *The teachers opened(PL) the bag in the garden but the bag was empty.*

4. CS-4: Mühendisler konuşan bir robot yaptı. *The engineers made a robot that can speak.*

NS-SG-4: Robotu dün tanıttı ve robot beğenildi. *(The engineers) introduced(SG) the robot yesterday and the robot was liked.*

NS-PL-4: Robotu dün tanıttılar ve robot beğenildi. *(The engineers) introduced(PL) the robot yesterday and the robot was liked.*

CS-4: Konuşan bir robot yapıldı. *A robot that can speak was made.*

OS-SG-4: Mühendisler robotu dün tanıttı ve robot beğenildi. *The engineers introduced(SG) the robot yesterday and the robot was liked.*

OS-PL-4: Mühendisler robotu dün tanıttılar ve robot beğenildi. *The engineers introduced(PL) the robot yesterday and the robot was liked.*

5. CS-5: Doktorlar yeni bir ilaç geliştirdi. *The doctors developed a new medicine.*

NS-SG-5: İlacı dün denedi ve sonuç iyiydi. *(The doctors) tried(SG) the new medicine and the result was good.*

NS-PL-5: İlacı dün denediler ve sonuç iyiydi. *(The doctors) tried(PL) the new medicine and the result was good.*

CS-5: Yeni bir ilaç geliştirildi. *A new medicine was developed.*

OS-SG-5: Doktorlar ilacı dün denedi ve sonuç iyiydi. *The doctors tried(SG) the new medicine and the result was good.*

OS-PL-5: Doktorlar ilacı dün denediler ve sonuç iyiydi. *The doctors tried(PL) the new medicine and the result was good.*

6. CS-6: Öğrenciler pahalı bir kitap aldı. *The students bought an expensive book.*

NS-SG-6: Kitabı derste okudu ama kitap sıkıcıydı. *(The students) read(SG) the book during the lesson and the book was boring.*

NS-PL-6: Kitabı derste okudular ama kitap sıkıcıydı. *(The students) read(PL) the book during the lesson and the book was boring.*

CS-6: Pahalı bir kitap alındı. *An expensive book was bought.*

OS-SG-6: Öğrenciler kitabı derste okudu ama kitap sıkıcıydı. *The students read(SG) the book during the lesson and the book was boring.*

OS-PL-6: Öğrenciler kitabı derste okudular ama kitap sıkıcıydı. *The students read(PL) the book during the lesson and the book was boring.*

7. CS-7: Köylüler tarlada bir silah buldu. *The peasents found a gun in the field.*

NS-SG-7: Silahı polise verdi ve soruşturma başladı. *(The peasents) gave(SG) the gun to the police and an investigation started.*

NS-PL-7: Silahı polise verdiler ve soruşturma başladı. *(The peasents) gave(PL) the gun to the police and an investigation started.*

CS-7: Tarlada bir silah bulundu. *A gun was found in the field.*

OS-SG-7: Köylüler silahı polise verdi ve soruşturma başladı. *The peasents gave(SG) the gun to the police and an investigation started.*

OS-PL-7: Köylüler silahı polise verdiler ve soruşturma başladı. *The peasents gave(PL) the gun to the police and an investigation started.*

8. CS-8: İşçiler kahvaltı için börek aldı. *The workers bought börek for breakfast.*

NS-SG-8: Böreği kahvaltıda yedi ama börek kötüydü. *(The workers) ate(SG) the börek at breakfast but the börek tasted bad.*

NS-PL-8: Böreği kahvaltıda yediler ama börek kötüydü. *(The workers) ate(PL) the börek at breakfast but the börek tasted bad.*

CS-8: Kahvaltı için börek alındı. *Börek was bought for breakfast.*

OS-SG-8: İşçiler böreği kahvaltıda yedi ama börek kötüydü. *The workers ate(SG) the börek at breakfast but the börek tasted bad.*

OS-PL-8: İşçiler böreği kahvaltıda yediler ama börek kötüydü. *The workers ate(PL) the börek at breakfast but the börek tasted bad.*

9. CS-9: Avcılar dün bir ayı gördü. *Yesterday, the hunters saw a beer.*

NS-SG-9: Ayıyı ormanda vurdu ve ayı yaralandı. *(The hunters) shot(SG) the beer in the forest and the beer was injured.*

NS-PL-9: Ayıyı ormanda vurdular ve ayı yaralandı. *(The hunters) shot(PL) the beer in the forest and the beer was injured.*

CS-9: Dün bir ayı görüldü. *Yesterday, a beer was seen.*

OS-SG-9: Avcılar ayıyı ormanda vurdu ve ayı yaralandı. *The hunters shot(SG) the beer in the forest and the beer was injured.*

OS-PL-9: Avcılar ayıyı ormanda vurdular ve ayı yaralandı. *The hunters shot(PL) the beer in the forest and the beer was injured.*

10. CS-10: Şoförler dün yeni arabayı denedi. *The drivers tested the new car yesterday.*

NS-SG-10: Arabayı çok beğendi ama araba pahalıydı. *(The drivers) liked(SG) the car but it was expensive.*

NS-PL-10: Arabayı çok beğendiler ama araba pahalıydı. *(The drivers) liked(PL) the car but it was expensive.*

CS-10: Dün yeni araba denendi. *The new car was tested yesterday.*

OS-SG-10: Şoförler arabayı çok beğendi ama araba pahalıydı. *The drivers liked(SG) the car but it was expensive.*

OS-PL-10: Şoförler arabayı çok beğendiler ama araba pahalıydı. *The drivers liked(PL) the car but it was expensive.*

11. CS-11: Politikacılar yeni bir kanun çıkardı. *Politicians introduced a new law.*

NS-SG-11: Kanunu halka açıkladı ama halk beğenmedi. *(The politicians) announced(SG) the law, but the public didn’t like it.*

NS-PL-11: Kanunu halka açıkladılar ama halk beğenmedi. *(The politicians) announced(PL) the law, but the public didn’t like it.*

CS-11: Yeni bir kanun çıkarıldı. *A new law was introduced*

OS-SG-11: Politikacılar kanunu halka açıkladı ama halk beğenmedi. *The politicians announced(SG) the law, but the public didn’t like it.*

OS-PL-11: Politikacılar kanunu halka açıkladılar ama halk beğenmedi. *The politicians announced(PL) the law, but the public didn’t like it.*

12. CS-12: Gazeteciler dün başbakanı ziyaret etti. *The journalists visited the president yesterday.*

NS-SG-12: Başbakana soru sordu ama başbakan cevaplamadı. *(The journalists) asked(SG) a question but he didn’t answer.*

NS-PL-12: Başbakana soru sordular ama başbakan cevaplamadı. *(The journalists) asked(PL) a question but he didn’t answer.*

CS-12: Dün başbakan ziyaret edildi. *The president was visited yesterday.*

OS-SG-12: Gazeteciler başbakana soru sordu ama başbakan cevaplamadı. *The journalists asked(SG) a question but he didn’t answer.*

OS-PL-12: Gazeteciler başbakana soru sordular ama başbakan cevaplamadı. *The journalists asked(PL) a question but he didn’t answer.*

13. CS-13: Seyirciler konser için bilet aldı. *The spectators bought tickets for the concert.*

NS-SG-13: Konser salonuna gitti ve konser başladı. *(The spectators) went(SG) to the concert area and the concert began.*

NS-PL-13: Konser salonuna gittiler ve konser başladı. *(The spectators) went(PL) to the concert area and the concert began.*

CS-13: Konser için bilet alındı. *Tickets were bought for the concert.*

OS-SG-13: Seyirciler konser salonuna gitti ve konser başladı. *The spectators went(SG) to the concert area and the concert began.*

OS-PL-13: Seyirciler konser salonuna gittiler ve konser başladı. *The spectators went(PL) to the concert area and the concert began.*

14. CS-14: Hırsızlar dün bir kuyumcuyu soydu. *The robbers robbed a jewelry store yesterday.*

NS-SG-14: Kuyumcuyu rehin aldı ama kuyumcu kurtuldu. *(The robbers) took(SG) the jeweler hostage but the jeweler escaped.*

NS-PL-14: Kuyumcuyu rehin aldılar ama kuyumcu kurtuldu. *(The robbers) took(PL) the jeweler hostage but the jeweler escaped.*

CS-14: Dün bir kuyumcu soyuldu. *A jewelry store was robbed yesterday.*

OS-SG-14: Hırsızlar kuyumcuyu rehin aldı ama kuyumcu kurtuldu. *The robbers took(SG) the jeweler hostage but the jeweler escaped.*

OS-PL-14: Hırsızlar kuyumcuyu rehin aldılar ama kuyumcu kurtuldu. *The robbers took(PL) the jeweler hostage but the jeweler escaped.*

15. CS-15: Dalgıçlar denizin dibinde hazine buldu. *The divers found a treasure at the bottom of the sea.*

NS-SG-15: Hazineyi karaya çıkardı ama hazine çalındı. *(The divers) took(SG) the treasure to the land but the treasure was stolen.*

NS-PL-15: Hazineyi karaya çıkardılar ama hazine çalındı. *(The divers) took(PL) the treasure to the land but the treasure was stolen.*

CS-15: Denizin dibinde hazine bulundu. *A treasure was found at the bottom of the sea.*

OS-SG-15: Dalgıçlar hazineyi karaya çıkardı ama hazine çalındı. *The divers took(SG) the treasure to the land but the treasure was stolen.*

OS-PL-15: Dalgıçlar hazineyi karaya çıkardılar ama hazine çalındı. *The divers took(PL) the treasure to the land but the treasure was stolen.*

16. CS-16: Hemşireler küçük çocuğu yatağa yatırdı. *The nurses put the little child to bed.*

NS-SG-16: Çocuğa iğne yaptı ve çocuk uyudu. *(The nurses) gave(SG) the child an injection and the child slept.*

NS-PL-16: Çocuğa iğne yaptılar ve çocuk uyudu. *(The nurses) gave(PL) the child an injection and the child slept.*

CS-16: Küçük çocuk yatağa yatırıldı. *The little child was put to bed.*

OS-SG-16: Hemşireler çocuğa iğne yaptı ve çocuk uyudu. *The nurses gave(SG) the child an injection and the child slept.*

OS-PL-16: Hemşireler çocuğa iğne yaptılar ve çocuk uyudu. *The nurses gave(PL) the child an injection and the child slept.*

17. CS-17: Turistler halk pazarını ziyaret etti. *The tourists visited the market.*

NS-SG-17: Pazarı çok beğendi ama pazar pahalıydı. *(The tourists) liked(SG) the market but the market was very expensive.*

NS-PL-17: Pazarı çok beğendiler ama pazar pahalıydı. *(The tourists) liked(PL) the market but the market was very expensive.*

CS-17: Halk pazarı ziyaret edildi. *The market was visited.*

OS-SG-17: Turistler pazarı çok beğendi ama pazar pahalıydı. *The tourists liked(SG) the market but the market was very expensive.*

OS-PL-17: Turistler pazarı çok beğendiler ama pazar pahalıydı. *The tourists liked(PL) the market but the market was very expensive.*

18. CS-18: Taksiciler benzin zammına karşı çıktı. *The taxi drivers opposed the increase in petrol prices.*

NS-SG-18: Zammı protesto etti ama zam değişmedi. *(The taxi drivers) protested(SG) the increase but the increase didn’t change.*

NS-PL-18: Zammı protesto ettiler ama zam değişmedi. *(The taxi drivers) protested(PL) the increase but the increase didn’t change.*

CS-18: Benzin zammına karşı çıkıldı. *The increase in petrol prices was opposed.*

OS-SG-18: Taksiciler zammı protesto etti ama zam değişmedi. *The taxi drivers protested(SG) the increase but the increase didn’t change.*

OS-PL-18: Taksiciler zammı protesto ettiler ama zam değişmedi. *The taxi drivers protested(PL) the increase but the increase didn’t change.*

19. CS-19: İtfaiyeciler yangın yerine hemen geldi. *The firefighters immediately arrived at the fire scene.*

NS-SG-19: Yangına müdahele etti ve yangın söndürüldü. *(The firefighters) responded(SG) to the fire and it was put out.*

NS-PL-19: Yangına müdahele ettiler ve yangın söndürüldü. *(The firefighters) responded(PL) to the fire and it was put out.*

CS-19: Yangın yerine hemen gelindi. *(The passive version in English is not possible)*

OS-SG-19: İtfaiyeciler yangına müdahele etti ve yangın söndürüldü. *The firefighters responded(SG) to the fire and it was put out.*

OS-PL-19: İtfaiyeciler yangına müdahele ettiler ve yangın söndürüldü. *The firefighters responded(PL) to the fire and it was put out.*

20. CS-20: Pilotlar uçakta bir sorun saptadı. *The pilots detected a problem on the plane.*

NS-SG-20: Uçağı alana indirdi ve uçak boşaltıldı. *(The pilots) landed(SG) the plane and the plane was disembarked.*

NS-PL-20: Uçağı alana indirdiler ve uçak boşaltıldı. *(The pilots) landed(PL) the plane and the plane was disembarked.*

CS-20: Uçakta bir sorun saptandı. *A problem was detected on the plane.*

OS-SG-20: Pilotlar uçağı alana indirdi ve uçak boşaltıldı. *The pilots landed(SG) the plane and the plane was disembarked.*

OS-PL-20: Pilotlar uçağı alana indirdiler ve uçak boşaltıldı. *The pilots landed(PL) the plane and the plane was disembarked.*

21. CS-21: Hostesler yolculuk sırasında yemeği ısıttı. *The flight attendants heated the food during the journey.*

NS-SG-21: Yemeği servis etti ama yemek kötüydü. *(The flight attendants) served(SG) the food but the food was bad.*

NS-PL-21: Yemeği servis ettiler ama yemek kötüydü. *(The flight attendants) served(PL) the food but the food was bad.*

CS-21: Yolculuk sırasında yemek ısıtıldı. *The food was heated during the journey.*

OS-SG-21: Hostesler yemeği servis etti ama yemek kötüydü. *The flight attendants served(SG) the food but the food was bad.*

OS-PL-21: Hostesler yemeği servis ettiler ama yemek kötüydü. *The flight attendants served(PL) the food but the food was bad.*

22. CS-22: Sanatçılar çocuklar için kampanya başlattı. *The artists started a campaign for children.*

NS-SG-22: Kampanyayı televizyonda açıkladı ve para toplandı. *(They) announced(SG) the campaign on TV and money was collected.*

NS-PL-22: Kampanyayı televizyonda açıkladılar ve para toplandı. *(They) announced(PL) the campaign on TV and money was collected.*

CS-22: Çocuklar için kampanya başlatıldı. *A campaign for children was started.*

OS-SG-22: Sanatçılar kampanyayı televizyonda açıkladı ve para toplandı. *They announced(SG) the campaign on TV and money was collected.*

OS-PL-22: Sanatçılar kampanyayı televizyonda açıkladılar ve para toplandı. *They announced(PL) the campaign on TV and money was collected.*

23. CS-23: Çalışanlar bugün yeni müdürle tanıştı. *The workers met the new manager today.*

NS-SG-23: Müdüre çiçek verdi ve müdür sevindi. *(The workers) gave(SG) flowers to the new manager and he was glad/happy.*

NS-PL-23: Müdüre çiçek verdiler ve müdür sevindi. *(The workers) gave(PL) flowers to the new manager and he was glad/happy.*

CS-23: Bugün yeni müdürle tanışıldı. *The new manager was met today.*

OS-SG-23: Çalışanlar müdüre çiçek verdi ve müdür sevindi. *The workers gave(SG) flowers to the new manager and he was glad/happy.*

OS-PL-23: Çalışanlar müdüre çiçek verdiler ve müdür sevindi. *The workers gave(PL) flowers to the manager and he was glad/happy.*

24. CS-24: Oyuncular yeni gösteri için hazırlandı. *The performers made preparations for the new show.*

NS-SG-24: Gösteriyi sahnede sergiledi ve gösteri beğenildi. *(The performers) performed(SG) the new show and the new show was liked.*

NS-PL-24: Gösteriyi sahnede sergilediler ve gösteri beğenildi. *(The performers) performed(PL) the new show and the new show was liked.*

CS-24: Yeni gösteri için hazırlanıldı. *Preparations were made for the new show.*

OS-SG-24: Oyuncular gösteriyi sahnede sergiledi ve gösteri beğenildi. *The performers performed(SG) the new show and it was liked.*

OS-PL-24: Oyuncular gösteriyi sahnede sergilediler ve gösteri beğenildi. *The performers performed(PL) the new show and it was liked.*
